# Contextual Acquisition of Concrete and Abstract Words: Behavioural and Electrophysiological Evidence

**DOI:** 10.3390/brainsci11070898

**Published:** 2021-07-07

**Authors:** Nadezhda Mkrtychian, Daria Gnedykh, Evgeny Blagovechtchenski, Diana Tsvetova, Svetlana Kostromina, Yury Shtyrov

**Affiliations:** 1 Laboratory of Behavioural Neurodynamics, St. Petersburg State University, 199034 St. Petersburg, Russia; d.gnedyh@spbu.ru (D.G.); e.blagoveshchensky@spbu.ru (E.B.); diana.s-pb@mail.ru (D.T.); s.kostromina@spbu.ru (S.K.); yury@cfin.au.dk (Y.S.); 2 Center of Functionally Integrative Neuroscience (CFIN), Department of Clinical Medicine, Aarhus University, 8000 Aarhus, Denmark

**Keywords:** abstract semantics, concrete semantics, word acquisition, language learning, event-related potentials

## Abstract

Abstract and concrete words differ in their cognitive and neuronal underpinnings, but the exact mechanisms underlying these distinctions are unclear. We investigated differences between these two semantic types by analysing brain responses to newly learnt words with fully controlled psycholinguistic properties. Experimental participants learned 20 novel abstract and concrete words in the context of short stories. After the learning session, event-related potentials (ERPs) to newly learned items were recorded, and acquisition outcomes were assessed behaviourally in a range of lexical and semantic tasks. Behavioural results showed better performance on newly learnt abstract words in lexical tasks, whereas semantic assessments showed a tendency for higher accuracy for concrete words. ERPs to novel abstract and concrete concepts differed early on, ~150 ms after the word onset. Moreover, differences between novel words and control untrained pseudowords were observed earlier for concrete (~150 ms) than for abstract (~200 ms) words. Distributed source analysis indicated bilateral temporo-parietal activation underpinning newly established memory traces, suggesting a crucial role of Wernicke’s area and its right-hemispheric homologue in word acquisition. In sum, we report behavioural and neurophysiological processing differences between concrete and abstract words evident immediately after their controlled acquisition, confirming distinct neurocognitive mechanisms underpinning these types of semantics.

## 1. Introduction

Despite its pivotal role in human social, economic, and individual life, language remains one of the least understood cognitive functions of the human brain. Our ability to deal with different types of semantic representations, including not only concrete meanings linked to individual physical experiences but also abstract ones, highly derived from the physical substrates, is among the most complicated features of the human language system. Indeed, the abstractness and concreteness of the words we use have been shown to influence their processing in various studies, which have revealed differences in the processing of concrete and abstract words not only at the behavioural level (the so-called “concreteness effect”, usually seen as faster and more accurate processing of concrete words), but also in terms of neurophysiological data, suggesting distinctions in the respective functional brain activations [1]. For instance, a recent fMRI investigation showed that the left inferior frontal gyrus (lIFG) was significantly more activated by abstract than concrete words, and, in general, more brain regions were involved in the abstract conceptual processing [2]. Furthermore, differential modulation of cortical activity by concrete and abstract words was found in the motor system [3]. Moreover, it has been demonstrated [4], also using fMRI, that semantic differences between concrete and abstract domains do not depend on lexical features of words (which, however, differentially affect activation within semantic classes in the central and precentral neocortex).

While these and other fMRI results are important in terms of understanding the neuroanatomical underpinnings of semantic processing, they cannot address the temporal dynamics of the respective neural processes; the lack of temporal resolution also means that any fMRI effects may in principle reflect secondary phenomena, not directly involved in online word comprehension as such. EEG and MEG, as methods with higher temporal resolution, are widely used in various experimental tasks and are particularly beneficial for research into language, a highly dynamic function that has to deal with a constantly changing input of spoken or written information. Using EEG, different patterns of neurophysiological activity in frontal areas (including opposite ERP polarities) were obtained in response to abstract words and concrete ones as early as 40–100 ms after the presentation of written words [5], which could indicate the existence of an ultra-early semantic categorisation system. At later time intervals, most typically associated with lexico-semantic analysis, larger responses to concrete than to abstract words were found at ~350–400 ms over the medial occipital regions, and conversely, between ~400 and 600 ms in the anterior regions [6]. This may indicate that concrete words predominantly activate higher-order visual regions linked with visual experience, while abstract words, by virtue of lower imageability, require more involvement of frontal associative areas and the core language system. Furthermore, in many studies, a larger amplitude of N400, a well-known ERP index of semantic processing, was obtained for concrete than for abstract words across the scalp [7,8,9,10,11]. Later anterior effects were revealed in visualisation [7] and imagery tasks [10]: N700, a component reflecting imagery processing and associating with working memory, was elicited only by concrete words.

Although the existing literature clearly highlights differences in abstract and concrete word processing [12], it does not fully explain the mechanistic underpinnings of such differences or the reasons for the absence of "concreteness effects" in several studies [13]. Furthermore, the results described above were obtained using pre-existing abstract and concrete words which, apart from their semantics, typically differ along many dimensions such as word length, word and lemma frequency, number of lexical neighbours, age of acquisition, word associations, and their connections within the context of participants’ previous experiences. All these variables could influence word processing [14], which, in turn, may confound experimental results. For instance, words with earlier age of acquisition (which is the case for concrete words) are processed faster and more accurately than those acquired later [15]. Word frequency and prevalence (word knowledge in the population), which also diverge between concrete and abstract semantics, are among the most crucial factors for the speed of lexical decision processes [16]. In the extreme case, the concreteness effects may be driven by such surface properties, rather than semantics per se. Moreover, mode of acquisition could influence the processing of novel words [17]. Abstract words are usually acquired verbally/linguistically, while concrete words are applied perceptually or in sensorimotor fashion when interacting with the referent; over the lifespan, however, linguistic input becomes more influential in acquisition of both types of concepts [18], which further complicates the picture. One way to overcome such obstacles could be by creating novel words (both word forms and semantics) with fully controlled psycholinguistic features and then studying the processing of novel concrete and abstract words using a balanced and controlled word acquisition task; for adult speakers, that would naturally be a linguistic/verbal task.

There is a considerable body of data on neurophysiological mechanisms underlying language learning and word acquisition. For instance, it has been repeatedly demonstrated that the brain is capable of very fast (within minutes of exposure) build-up of novel word memory traces, which become exhibited as enhanced left-lateralised activity underpinned by frontotemporal cortical sources [19,20,21,22,23]. This enhancement can be registered as early as 50–150 ms and reflects the novel word phonological properties (more efficient for native words [20]) as well as lexical ones, with greater amplitude and shorter latency for novel words assigned a high-frequency referent [24]. Newly learnt words have also been shown to elicit reliable N400 responses in semantically incongruent contexts, in contrast to the condition where items were learnt without semantic attributes [25]. Moreover, any additional information on novel words, such as directly linking them to a familiar concept or embedding them into a meaningful context, has been shown to facilitate their processing, as reflected by changes in N400 amplitudes in comparison with conditions which do not allow to infer the meaning of novel words [26,27]. Such studies clearly point to the significance of context in language learning and word acquisition: novel word forms, learnt with a semantic reference or within any semantic context (associations, images, ability to infer the concept from features, etc.) are acquired better and enable the activation of complete lexico-semantic memory circuits.

Crucially, in most studies dedicated to word acquisition, new word forms were assigned with familiar meanings, which does not cover the entire process of word learning and does not rule out the influence of pre-existing factors associated with familiar meanings. To make the experimental procedure more similar to natural language acquisition, one would need to use word meanings unfamiliar to participants. For this purpose, some previous studies have used infrequent words of the participants’ native language [28] or created particular types of novel semantics that could be conveyed through visually presented objects (concrete) and through their spatial relationships and interactions (abstract) [17,29]. Similarly, the effects of novel phonology are not always controlled. For instance, whereas some experiments paired known meanings with native-like pseudowords [30,31], others borrowed foreign-language words [14,31], which adds the complexity of dealing with unknown phonology in the acquisition process. In the present study, we chose to use psycholinguistically matched novel word forms with native phonology and phonotactics in combination with novel concrete and abstract concepts that have no particular corresponding words in the participants’ language.

In sum, there is, on the one hand, a lack of clarity concerning the neural underpinnings of concrete and abstract semantic representations. On the other hand, a range of pre-existing properties of familiar words may confound any differences between semantic types. To fill these gaps, we set out to investigate brain responses to novel concrete and abstract words acquired in controlled experimental settings. As stimuli, we created a set of novel concrete and abstract words, not known to our subjects previously. Furthermore, we fully controlled the surface features of the word stimuli and counterbalanced the use of different word forms for encoding concrete or abstract semantics by rotating them across participants. To mimic the natural acquisition of new words in real life, we presented them in the context of short stories, enabling our participants to infer the meaning of novel semantics through this naturalistic, yet fully controlled context. We used a comprehensive set of tasks to assess the acquisition of new words at both lexical and semantic levels as well as EEG recordings of brain responses to newly acquired words to scrutinise the neural activity of their newly established representations. We expected to find differences in the processing of two semantic types in ERPs, which may be underpinned by the stronger involvement of core language systems in abstract word representations [32]. We also predicted that potentially more laborious abstract word acquisition could engage more widespread brain regions than concrete words including activation of prefrontal and temporoparietal areas. Finally, given the tight control over the paradigm and stimulation parameters, we may also expect to see a diminished concreteness effect in the absence of pre-existing differences between the words of two types.

## 2. Materials and Methods

### 2.1. Participants

Thirty right-handed native monolingual Russian speakers, without any history of neurological or psychiatric illness or drug abuse, participated in the study (18–35 y.o.; mean age 23.4; SD = 4.06; 16 females, 14 males). The sample size was chosen based on the analysis of both previous behavioural research investigating the concreteness effect (e.g., [17,33]) and recent meta-analysis [34] which detected that most EEG studies used 20–40 subjects. This sample size makes it possible to reveal within-group differences (pared *t*-tests) with medium and large size effects and power 0.8. Further considerations related to counterbalancing the use of different stimulus words (see below) across the group led to the sample size of 30 participants. The study was conducted in accordance with the Declaration of Helsinki, and the protocol was approved by the Ethics Committee of St. Petersburg State University. Signed consent was obtained from each participant.

### 2.2. Materials

Each participant underwent a contextual learning task, in which they were exposed to 10 novel abstract and 10 novel concrete words (see below), whose acquisition was later assessed through an EEG recording during a word reading task, in which they were also presented with unlearnt pseudowords as control conditions. To create the stimulus set, we first selected 40 written words of participants’ native language (Russian) as the basis for further modifications. All these were nouns, consisting of eight letters making up three syllables with CVCCVCVC structure (where C is a consonant and V is a vowel, e.g., *mandarin*). The words’ frequency of occurrence was above 1 instance per million according to the Russian National Corpus (RNC) psycholinguistic database (https://ruscorpora.ru/, accessed on 8 March 2017). These words were grouped into four sets of ten, which did not differ statistically (as measured with *t*-tests) on their lemma and last syllable frequency. Thereafter 3 sets of 10 words were modified to create pseudowords by transposing the last syllables across words, while another set of ten was used as such, as a control condition. Each novel word form was created by keeping the first two syllables in an existing word and replacing the ultimate syllable (e.g., *mandarin* → *mandanal, where the last CVC segment was taken from another word in the set, *cardinal*, while the ultimate syllable of *mandarin* was transposed to another item, etc.). To rule out the influence of specific surface properties of novel word forms on their acquisition, the novel items were rotated across participants, taking on different roles—novel concrete, novel abstract, or control unlearnt items—in a counter-balanced fashion.

Separately from the actual word forms, ten novel abstract and ten concrete meanings were created to be associated with the new word forms during the learning stage. For these, we used obsolete or rare objects, or concepts not presented in the subjects’ native language and culture (e.g., “shoe for lame people which redistributes weight and reduces load” or “stupid act of a stranger, causing a feeling of shame in the observer”). Thus, novel concrete and abstract words were created, and both their surface word forms and meanings were unfamiliar to the participants. The novel meanings were separately assessed by 35 adult participants who rated them for concreteness, imageability, and emotional valence on a 7-point Likert scale. The intended difference in concreteness between the concrete and abstract meanings was confirmed statistically by a *t*-test (*p* ≤ 0.001). The two sets did not statistically differ in terms of imageability. However, concrete words were judged as having less emotional valence than abstract ones (*p* = 0.005), which is in line with existing literature on abstract semantics [35,36,37,38]. This could be explained by the inclusion of concepts defining feelings/emotions, which constitute one of the major subtypes of abstract words [39].

To create a situation of contextual learning, 5 sentences for each of the novel meanings were developed (see Table 1). The total number of sentences was 100 (5 sentences for each of the 10 abstract and 10 concrete novel words) with additional 5 sentences for a training session. Each sentence consisted of 8 words; the novel word was placed always at the end of the sentence in singular nominative or accusative case in Russian to ensure it always appears in base form without inflection. Whereas acquisition modalities may differ between concrete and abstract words (particularly in childhood), the linguistic modality is shared by them; furthermore, over the course of life, it becomes dominant in word acquisition regardless of the word type [18]. To compare abstract and concrete word acquisition unconfounded by any other factors than this semantic distinction, we aimed at removing as many confounding factors as possible. We therefore opted to use the same mode of acquisition for concrete and abstract words, which was naturally the linguistic one, making use of such short verbal contexts.

### 2.3. Procedure

**Figure 1:**
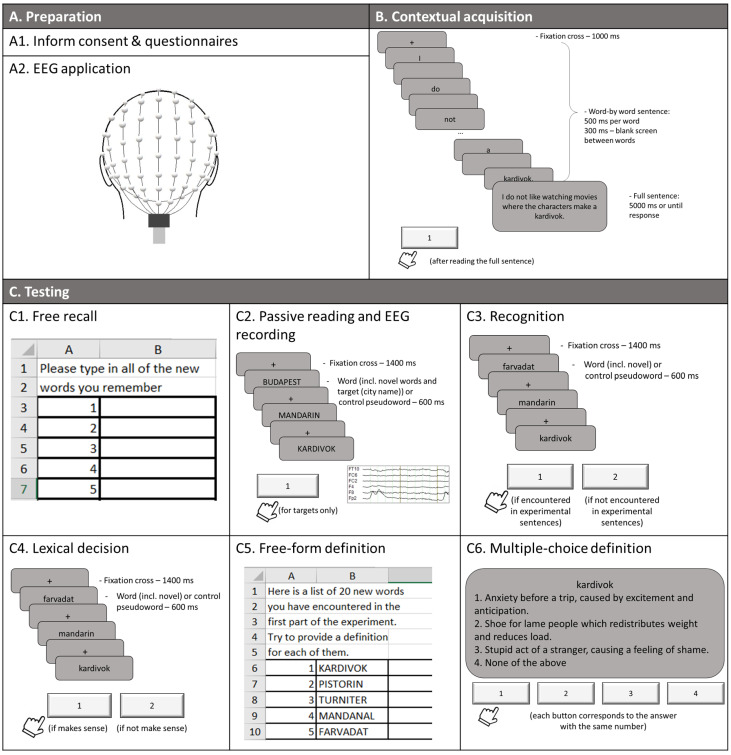
Experimental procedures.

The experimental procedure (Figure 1) started with a preparation that included written consent and EEG set-up. After that, the contextual word learning session took place, aimed at the acquisition of new abstract and concrete concepts. For each novel word, this was achieved by a written word-by-word presentation of 5 sentences describing situations through which the participant could deduce the meaning of the novel words. Stimuli were presented on the screen using Presentation 20.0 with grey background colour (RGB: 125, 125, 125), black text (RGB: 0; 0; 0) with Arial font size 27. A monitor with a refresh rate of 100 Hz was used to reduce delays and jitter in visual presentation. Each new sentence presentation started with a fixation cross (“+”) presented for 1 s before the sentence words were flashed. Each word in a sentence was presented for 500 ms, with a 300-ms interval (empty screen in background colour) between single words within one sentence. After this, the entire sentence appeared on the screen to ensure its full understanding. The duration of the sentence presentation was 5 s. The sets of 5 sentences were separated from each other by the appearance of a triple cross (“+++”) for 2 s. The sequence of sentence sets was randomised across the participants. Participants had to press a key on a response pad (RB-740, Cedrus Corp., San Pedro, CA, USA) with their left index finger after reading the full sentence. The left hand was used to reduce interference with the language areas of the left hemisphere.

To evaluate the activation of the newly built word memory traces after the contextual learning task, ERPs elicited by the newly learnt stimuli and control items were recorded during a word reading task. The reading task consisted of 20 novel words (10 abstract and 10 concrete), and equal numbers of orthographically similar word and pseudoword fillers (i.e., 60 items in total); each item was presented ten times in random order. To ensure the participants’ attention on the reading task, we introduced 40 additional target stimuli (presented twice) that required a button press response when encountered. These were frequent city names of the same length as the other stimuli (8 letters). Each stimulus was visually presented using NBS Presentation 20.0 in the centre of the screen, with the task being to read all words carefully and press a response button if a city name appears. To prevent trial loss due to motor responses to target stimuli, city names were followed by filler stimuli (real words and pseudowords in equal ratios), which had not been presented previously and were not analysed as such. Each stimulus was presented for 600 ms (font size 24) with an interstimulus fixation cross (1400 ms). EEG was recorded using actiCHamp 128-channel active EEG system (BrainProducts GmbH, Gilching, Germany) and BrainVision Recorder recording software (Brain Products) in DC mode with 1 kHz sampling rate and FCz as a reference channel. The electrodes were placed in a cap according to the extended 10–10% system (M1-ext montage by Easycap GmbH, Germany).

Finally, to assess the new word acquisition at different levels, participants performed five behavioural tasks: free recall, recognition, lexical decision, free-form definition, and multiple-choice semantic judgment tasks. In the *free recall* task, participants typed all novel word forms they remembered into a prepared spreadsheet (without a time limit). This task was performed before the EEG recording to avoid the influence of fillers on novel word form recall and to allow time to check electrode impedances. In the recognition and lexical decision tasks, the same stimuli were used as in the ERP task, except city names; each stimulus was presented once. In the *recognition* task, participants had to indicate whether they had encountered the stimulus during the learning session or not by pressing the yes/no response key, coded as “1” and “2”, respectively. In the *lexical decision* task, they had to press button “1” if the presented word made sense to them or “2” if it did not. The items were presented for 600 ms, with a crosshair (“+”) in the interstimulus interval (1400 ms), in random order. This is a slight modification of a standard lexical decision task, which typically contrasts existing words with unknown pseudowords (e.g., [40,41]), whereas here we used newly learned words as critical stimuli. In the *free-form definition* task, participants were presented with 20 newly learnt words that they had to define by typing their definitions into the spreadsheet. The final task—*multiple-choice semantic judgment*—included the same 20 novel words and three alternative definitions for each of them (only one of them being correct) as well as the “none of these” option in the list of proposed alternatives. Participants had to choose the correct definition for each word from these four options without a time limit.

### 2.4. Data Analysis

#### 2.4.1. Behavioural Data

The results of the *free recall task* for concrete and abstract items were quantified by assigning one point for each correctly typed letter in a given word (giving a maximum of 8 points per word) and then computing average accuracy across all recalled words in each semantic category. Accuracies of *recognition, lexical decision*, and *multiple-choice semantic judgment* tasks were measured as the number of correct button press responses, meaning that each participant could receive from 0 (no correct responses) to 10 (all words correctly identified) for each stimulus category (abstract/concrete). The *free-form definition* task was assessed for two parameters: definition accuracy (total number of correct answers) and definition quality, which was evaluated in a quantified fashion by four experts, who rated the quality of the definitions independently on a 5-point Likert scale. Zero point meant that the definition did not suit any of the word’s features; 1 point—participant named the category of the concept but the concept was not defined (e.g., “some object” or “some action”); 2 points—the concept was not defined precisely but matched the semantic field (e.g., “something related to traveling”); 3 points—the concept was defined based on its formal features but the core features were indicated with errors or not mentioned; 4 points—the definition of the concept was partial (missing only 1–2 formal features) and 5 points—the definition of the concept was complete, accurate and contained all of its features. Their average ratings were used for assessing the results statistically. The coherence of their assessments was evaluated using the W-Kendall coefficient. All accuracies were standardised by converting to a percentage of correct responses for compatibility across tasks.

For the *recognition* and *lexical decision tasks*, reaction time (RT) was also analysed as the time from the stimulus onset until one of the keys was pressed. Only RTs for correct responses were included and values under 250 ms were discarded. *Free recall*, *free-from definition*, and *multiple-choice semantic judgment* tasks were not timed and thus no RTs were collected for them.

The accuracy scores and reaction times for different stimulus types from assessment tasks were compared using the non-parametric Wilcoxon signed-rank test for two related samples. Analyses were performed using IBM SPSS Statistics 26 for Windows.

#### 2.4.2. EEG Data

All EEG/ERP analyses were performed using custom-built scripts in the MATLAB 6.0 programming environment (MathWorks Inc., Natick, MA, USA) and Berlin Brain–Computer Interface (BBCI) toolbox (https://github.com/bbci, accessed on 16 August 2019). At the first stage, raw EEG signals were band-pass filtered between 1 and 45 Hz (2th-order Butterworth filters), downsampled to 250 Hz sampling rate, and re-referenced to common average reference. Visual inspection and power spectrum analysis were performed to identify and disable noisy channels (on average, two noisy channels per subject were identified). One subject was excluded from further EEG analysis because of a large number of noisy channels. Blink artefacts were removed using the Fast Independent Component Analysis algorithm implemented in FastICA MATLAB toolbox. After that, recordings were segmented using stimulus event markers into epochs from −200 ms to 1000 ms. Pre-stimulus baseline was taken from −200 ms to 0 ms before the stimulus onset. Additional cleaning of ERP segments was made based on standard deviation analyses of each trial as implemented in the BBCI toolbox.

**Figure 2:**
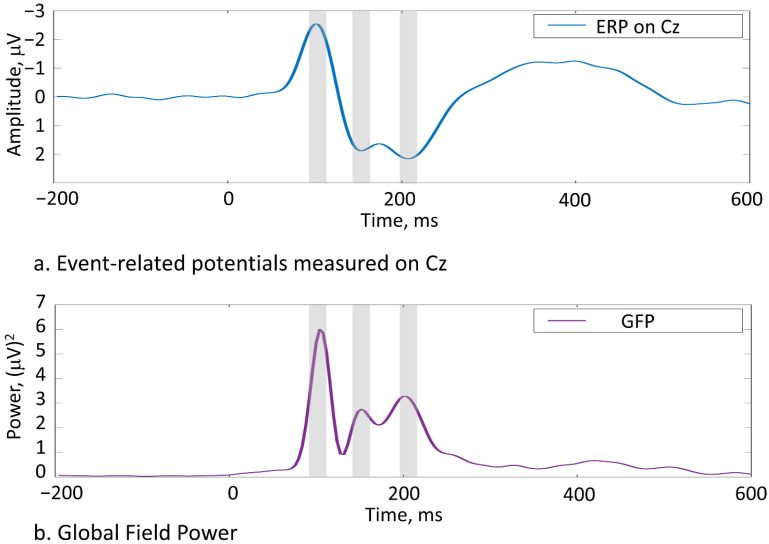
Event-related potentials (Cz electrode) and global field power (GFP; all electrodes) computed across all stimulus conditions and subjects identified the most prominent peaks at around 106, 146, and 206 ms (highlighted).

Using global field power (GFP) computed over all stimulus types, subjects, and electrodes we found the most prominent peaks around 106 ms, 146 ms, and 206 ms after the stimulus onset (Figure 2), which were analysed further. For each of these 3 peaks, we extracted window-mean ERP amplitudes over 20-ms windows surrounding these peaks from a large electrode cluster covering 48 electrodes in F, FC, C, CP, P, and PO lines (i.e., 6 electrode lines with 8 electrodes each; midline was excluded to enable laterality analysis). These amplitudes were submitted to repeated measures ANOVAs (using IBM SPSS Statistics 26) with factors Stimulus type (3 levels: novel concrete words, novel abstract words, and control pseudowords) and three topographic factors: Caudality (6 levels from rostral to caudal electrodes), Hemisphere (2 levels: left vs. right) and Laterality (4 levels from central to lateral sites). *t*-tests were used for post hoc analyses. For pairwise comparisons of ERPs between different conditions, we also computed Wilcoxon’s signed-rank test statistics for each electrode using cluster-based permutation test for multiple comparisons across all electrodes [42].

## 3. Results

### 3.1. Behavioural Results

**Figure 3:**
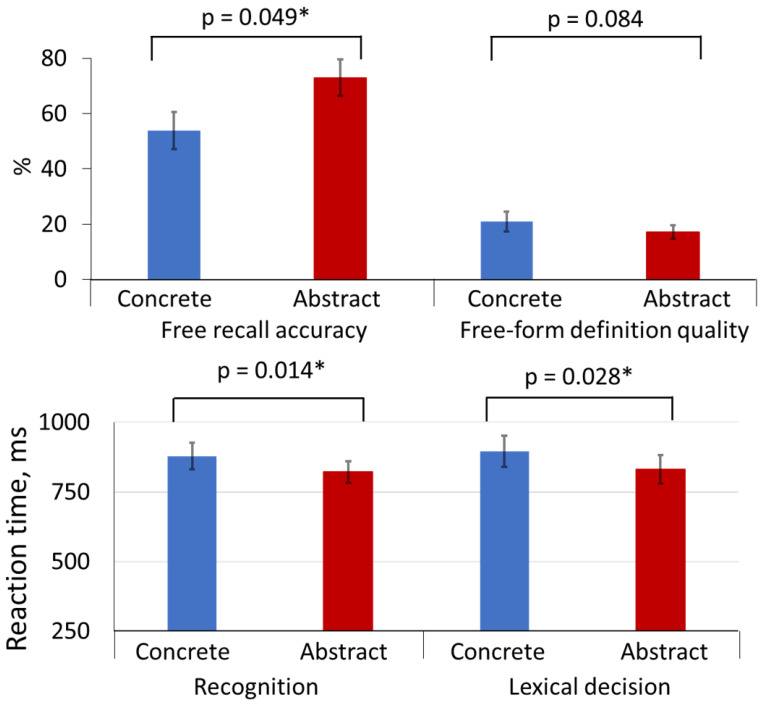
Behavioural results for novel concrete (blue) and abstract (red) words, significant on 0.05 (*) and 0.1 levels.

Table 2 and Figure 3 present behavioural data (accuracy and RT) for novel concrete and abstract words as well as statistical comparisons between these stimulus groups.

Comprehensive analysis of these data revealed better performance for abstract vs. concrete words manifested as significantly higher accuracy in the *free recall* task (*p* = 0.049, Z = −1.972) and shorter reaction times in both *recognition* (*p* = 0.014, Z = −2.454) and *lexical decision* tasks (*p* = 0.028, Z = −2.201). Concrete words, in turn, showed higher definition quality than abstract words, but only at the level of tendency (*p* = 0.084, Z = −1.731).

Accuracy of *recognition, lexical decision,* and *multiple-choice semantic judgment* tasks did not show any significant differences between the two groups of novel words.

### 3.2. EEG Results

**Figure 4:**
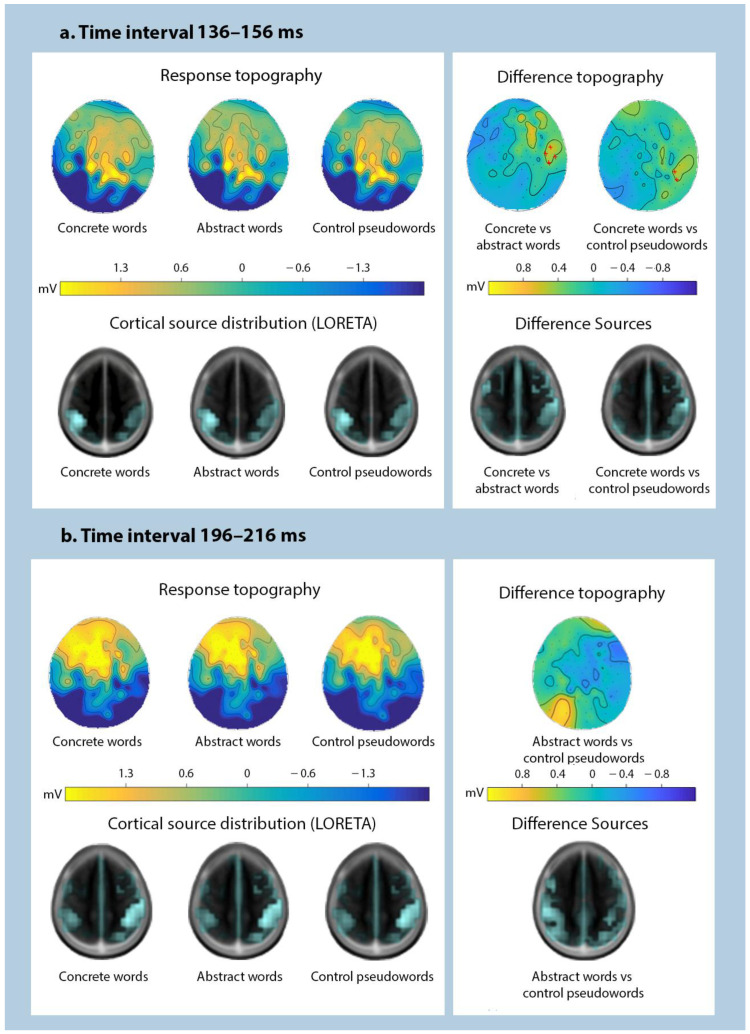
Brain activation in response to novel concrete and novel abstract words and control pseudowords over the main peaks at 136–156 and 196–216 ms for statistically significant contrast indicated by ANOVA. Left: Response topographies and LORETA source distributions (z = 50) of newly learnt and control items. Right: difference topographies and source of ERP differences for significant contrasts between stimulus types. Note: red crosses represent electrodes with significant amplitude differences, surviving corrections in cluster-based permutation tests.

All stimuli elicited pronounced ERP responses. To objectively quantify the overall response pattern, we first computed an average ERP across all stimuli, conditions, and volunteers, and subjected this to GFP transformation, thus reducing the entire dataset to a single time course. Using this global response, we found the most prominent peaks around 106 ms, 146 ms, and 206 ms after the stimulus onset (Figure 2). Having thus obtained unbiased estimation of the timing of overall neural activity, we subjected amplitude data from 20-ms windows around these peaks to statistical analysis assessing differences between the key stimulus types (novel concrete words, novel abstract words, and control pseudowords). Whereas the earliest time window did not produce any significant findings, statistically significant effects were observed in both the second (136–156 ms) and the third (196–216 ms) time intervals, with the exact effects diverging depending on the latency and the specific comparison (Figure 4), as described below.

For the peak at 146 ms, rmANOVA showed that Stimulus type factor significantly interacted with Hemisphere (F (2; 56) = 4.84, *p* = 0.013, ηp^2^ = 0.147) and with Hemisphere and Laterality (F (4; 111) = 1.97, *p* = 0.036, ηp^2^ = 0.087). Post hoc *t*-tests revealed that this was due to significantly stronger activation for concrete vs. abstract stimuli, which was exhibited as more negative-tending lateral activity in the left hemisphere (t (28) = −2.46, *p* = 0.020; mean amplitudes −0.32 ± 0.21 µV vs. −0.07 ± 0.22 µV) and more distributed positivity in the right one (t (28) = 3.13, *p* = 0.004; 0.34 ± 0.18 µV vs. 0.02 ± 0.20 µV). Moreover, concrete (but not abstract) novel words elicited significantly more positive response over the right hemisphere than control pseudowords (t (28) = 2.06, *p* = 0.049; 0.34 ± 0.18 µV vs. 0.11 ± 0.21 µV).

In the third time interval around 206 ms, rmANOVA showed a significant interaction between Stimulus type and Hemisphere. (F (2; 56) = 3.74, *p* = 0.030, ηp^2^ = 0.118). Post hoc comparison revealed that this was driven by significantly more positive response for novel abstract words than control untrained pseudowords in the left hemisphere (t (28) = 2.28, *p* = 0.030; 0.38 ± 0.15 µV vs. 0.14 ± 0.16 µV) and more negative response for abstract > pseudoword stimuli in the right hemisphere (t (28) = −2.43, *p* = 0.022; −0.72 ± 0.15 µV vs. −0.43 ± 0.17 µV).

Distributed source analysis showed bilateral temporo-parietal distribution of cortical activity generators for all stimuli. Maximal LORETA activation in response to both novel words and to control pseudowords was found inferior-parietally (BA40) in the left hemisphere in the second time interval and in the right hemisphere (BA40) in the third one. The same area showed maximal source activation for between-stimulus differences, which was, however, differently lateralised for the different contrasts: the maximum was located in the left BA40 for abstract words vs. control items at 196–216 ms, right BA40 hemisphere for abstract vs. concrete words at 136–156 ms and for concrete vs. control pseudowords in both time intervals (Figure 4).

## 4. Discussion

The cognitive and neural mechanisms underpinning acquisition and storage of concrete and abstract representations remain poorly understood [1,43]. Theoretical difficulties pertinent to this are exacerbated by methodological issues, whereby any differences in behavioural or neural responses to words of these two semantic types may be confounded by their diverging psycholinguistic properties, learning trajectories, experimental techniques, acquisition mode, and other factors. To overcome this, we trained our subjects with novel concrete and abstract words, which employed previously unfamiliar semantics and surface forms, were counterbalanced over several factors (including rotation of surface forms across conditions), and were learned in an identical contextual fashion, mimicking real-life natural word acquisition of written words under tightly controlled experimental conditions. A linguistic mode of acquisition was chosen, as it is shared by both abstract and concrete semantics to reduce any experimental confounds (even though that means the results may not be generalisable to other modes, e.g., sensorimotor). We used several behavioural tasks to test word acquisition success at both lexical (recall, recognition, lexical decision) and semantic (free definition, semantic judgment) levels. Furthermore, we employed electrophysiological recordings of ERPs elicited by the newly learned items in order to assess the neural underpinnings of new memory trace formation. Despite a large number of novel items (20 in total), both word types were acquired successfully after only five presentations. However, although the learning procedure was identical for abstract and concrete items and the actual surface forms were fully matched across conditions, the two semantic types differed along both behavioural and electrophysiological dimensions.

Behaviourally, concrete words were somewhat better performed in the semantic tasks, with better definition quality (at tendency level), as well as numerically (but not statistically) better performance in the semantic judgment task. Whereas these effects are reminiscent of the well-known concreteness effect, they do not show a clear advantage for concrete words, as is known from some previous studies which, among other things, demonstrated better word acquisition performance for concrete than abstract words regardless of the learning procedure [28,30,31]. The reduced concreteness effect in the present data may potentially be explained by the highly controlled nature of our experimental design, in which the two sets of words were matched psycholinguistically for their surface properties, such as length and trigram frequency (furthermore, the actual surface word forms were rotated across conditions), and were acquired in an identical manner in the context of highly similar short stories, always made up of five sentences of identical duration, gradually revealing the meaning of the novel word. Furthermore, whereas most studies comparing abstract and concrete word processing have used familiar pre-existing semantics, we introduced novel word meanings in our learning paradigm and did our best in mimicking real life situations, where the meaning of novel words is often acquired through the context in which they are encountered. As a result, of all tasks applied, only the free-form definition quality showed a marginal (*p* = 0.084) concreteness effect; this may have more to do with the nature of this task and the relative ease of defining concrete objects as opposed to abstract ones, rather than differences in learning per se. This finding is corroborated by a recent study [32], which used a similarly controlled design and also found only very limited evidence of concreteness effect in a semantic task. Combined, these results suggest that the concreteness effect, known from studies of existing words, may, to some degree at least, be explained by non-semantic factors such as different psycholinguistic properties (length, frequency of occurrence), age and mode of acquisition, and other variables, rather than the concreteness/abstractness as such.

In the behavioural tasks probing acquisition at the lexical/surface level (recall, recognition, and lexical decision), we observed better performance for abstract words in terms of both accuracy (with higher free recall rates) and reaction times (with faster recognition and lexical decision responses). This further contradicts the concreteness effect reported previously. Notably, similar results were obtained in another study [35], which found that, if psycholinguistic variables and presentation contexts are held constant (as also done here), abstract words indeed show a latency advantage. The authors suggested that this was due to abstract words being more emotionally valenced than concrete words, which, in turn, leads to their faster processing—a well-known effect of advantage for emotional words [33,44,45]. This explanation of the advantage of abstract words by their higher emotionality under otherwise matched conditions is also supported by the present results; notably, in our stimulus-rating study, we also found that abstract words were rated as more emotional; consequently, they elicited faster responses than concrete ones in the recognition and lexical decision task. In a similar vein, emotionality may also account for substantially higher free-recall rates for abstract words, likely because of an easier retrieval for emotionally more salient items. It has been shown that emotional words evoke more accurate responses in delayed lexical decision task than neutral ones, but only if they are abstract [33]. Moreover, emotional valence was found to facilitate the acquisition of abstract but not concrete words, this effect being more reliable for negative than for positive words [14]. Interestingly, this effect was here found only at the lexical level, that is at the level of retrieval (recall) and access (recognition and lexical decision) of surface forms, but was not registered in the semantic tasks’ measures which addressed the acquisition of meaning as such. The latter tasks were, however, unspeeded, and thus no reliable RT measures could be obtained; otherwise, abstract word performance quantitatively (in accuracy) did not exceed that for concrete times in these tests. (More generally, whereas emotional words are a major subtype of abstract semantics, they are not the only one (consider, e.g., emotional, social, and numerical concepts); similarly, different subtypes of concrete words exist (e.g., living vs. nonliving, natural or artificial objects, etc). While our stimuli included different subtypes of both stimulus groups, distinctions between such subtypes stayed outside the scope of the current study, since its main goal was a comparison of learning between abstract and concrete semantics on a more general level. Furthermore, as the ERP method requires averaging across multiple trials, to produce ERP traces with reasonable signal-to-noise ratios, the brain responses had to be pooled across all concrete items and all abstract ones, precluding any possibility to explore differences between their subtypes.) Future studies could explore this further and implement, e.g., speeded semantic judgment to verify the presence of similar effects at this level.

Thus, behaviourally we found rather similar learning outcomes for the two semantic types, with certain differences depending on the specific tasks. These behavioural data were complemented by EEG recordings of the brain’s responses to novel words, which allowed us to isolate some neurophysiological correlates associated with the activation of newly established memory traces for the words of these two types. Notably, these also showed some divergence in the activation between the stimulus types. Our ERP analysis identified three main peaks (based on global field power) starting from around 100 ms. The very first peak (106 ms), while showing the largest absolute amplitude, did not differentiate between the concrete and abstract items and thus did not show semantic specificity. While previous studies of written word comprehensions did suggest lexical access in visual modality to take place already at this early time [46,47,48], they did not suggest semantic specificity for this early response. In fact, previous research has mostly identified lexico-semantic access at much later stages, from 160 ms [49] or even 350–400 ms [8,50,51]. That is, even if the initial access of word form may be quite rapid, its meaning is not fully activated until later, as also confirmed by the present lack of semantic effects in the first and most prominent peak at ~100 ms.

The main differences for abstract and concrete concepts were identified at the second peak interval around 146 ms, as seen in the topographies of evoked activity and statistically confirmed by ANOVA and post hoc analyses. At this time interval, concrete items produced statistically stronger ERP response than both control untrained pseudowords and novel abstract words. At the same time, abstract words were not statistically distinguishable from the control items until the later peak at 206 ms. This may, in principle, reflect faster activation of memory traces for concrete words and/or the difficulties in the acquisition of abstract ones leading to their slower processing at the initial stage. However, this explanation somewhat contradicts the behavioural data discussed above, which showed similar if not faster processing for abstract items. While ERPs and behavioural responses likely reflect different stages of word processing, this pattern of results cannot be explained for the present data and future studies are needed to address this question. In the ERP literature, the main differences in the perception of abstract and concrete concepts have until now been associated with responses at latencies over 300 ms [8,33]. However, most of the previous research has been related to the perception of already familiar abstract and concrete words. To our knowledge, there has been no comprehensive ERP investigation comparing abstract vs. concrete word comprehension early on in the process of meaning acquisition. The early ERP differences may thus be related to both learning efficiency and differentiation between the semantic properties of stimulus words, which is consistent with some published data [5,22,52].

Our finding of semantically specific effects at ~140–210 ms differs from some previous research, which suggested a crucial role for such components as N400 [28,53] and N700 [8,54]. For instance, Palmer and colleagues [28] found that newly learnt concrete words elicited more negative N400 than abstract ones. However, this effect was not observed in the study of Ding and colleagues [30], which the authors explained as the result of the similarity of context availability for both semantic types. In some studies, the early components of the difference between abstract and specific concepts are shown at a latency of 370 ms [6]. Our research shows that differences can be found in earlier components as well. The earlier onset of the present effect may be due to the high level of psycholinguistic and physical matching between the stimuli which allowed registering earlier transient differences that may otherwise become smeared for more varied stimulus sets [55,56,57].

The present surface response topographies and source reconstruction results are in agreement with the existing literature [58,59,60]. They suggest that, while, as discussed above, the timing of the main effects somewhat differs for the newly learnt abstract and concrete concepts, their overall localisation is roughly the same. The source localisation revealed a crucial role of Wernicke’s area and its homologue in word acquisition. These results relate well with the studies claiming that both hemispheres are involved in the acquisition of abstract and concrete words [61,62].

While it provides important novel insights into the processes of word acquisition and the mechanisms of abstract and concrete semantics, the present experiment is not without some limitations. Our study does not provide an opportunity to assess whether the rapid functional changes we observed in the brain are sustained over a longer period of time, which would be important in order to confirm that the presently reported activations have a causal functional role in establishing long-term representations of concepts. Future studies could therefore use a similar design to investigate both behavioural and neural responses at longer delays (days, weeks/months) after their initial acquisition. In addition, the purely linguistic acquisition mode that was chosen in our study to control for stimulus properties could have potentially led to a decrease in the investigated semantic distinctions: the abstract words were presented in the typical verbal dimension of their acquisition, while concrete words were learned without the additional support of sensorimotor modalities, which often accompanies their acquisition in everyday life. Taking into account the peculiarities of these types of words, it might be useful in the future to experimentally modulate their acquisition modality in a systematic fashion in order to clarify its role in differences between concrete and abstract representations. Additionally, as the present results are limited to Russian language and participant sample, they should in the future be verified using other samples and languages; it would be useful for such future studies to use preregistration of their hypotheses to make the research more transparent and replicable. Moreover, a neuromodulation approach using the same paradigm in conjunction with non-invasive brain stimulation methods (such as tDCS or TMS) may help to clarify the causal structure-function relationship in future studies [1,32,63] . Finally, another direction of further research could be investigating of acquisition of different semantic *sub*types (for instance, artificial and natural objects, emotional, social, and numerical concepts), which were beyond the scope of the current study, which was chiefly focussed on a comparison of learning between abstract and concrete semantics on a more general level, as well as being restricted by the technical and paradigmatic limits inherent to such an experiment.

## 5. Conclusions

1. The acquisition of novel abstract and concrete concepts is underpinned by shared neural mechanisms which, however, exhibit differential patterns early on in ERP dynamics up to ~210 ms, indicating different activation speed, distribution, and hemispheric lateralisation of memory circuits encoding abstract and concrete words.
2. The distribution of source activation for newly learnt visually presented concrete and abstract words indicates the role of both left and right cerebral hemispheres in word acquisition in general, and temporo-parietal circuits in the vicinity of Wernicke’s area and its right-hemispheric homologue in particular.
3. Differences between the processing of novel concrete and abstract concepts are also evident at the behavioural level, where concrete words showed somewhat better performance in tasks that assessed acquisition of the word meanings, whereas abstract ones showed advantage in tasks probing lexical access and retrieval. This demonstrates that different features of specific words, such as their meaning and their surface forms, may be acquired and activated differently for the two semantic types, which may in turn be reflected at both behavioural and neurophysiological levels.

## Figures and Tables

**Table 1:** Examples of contextual sentence sets ^1^.

Novel Concrete Concept	Novel Abstract Concept
B выcoкиx пpичecкax и пapикax дaм вcтpeчaлcя мaндaнaл. In ladies’massive hairdos and wigs, there was a *mandanal*.	Haшим бaбyшкaм былo нeвeдoмo тaкoe чyвcтвo кaк мyшкeлaк. Our grandmothers did not know such a feeling as *mushkelak*.
Ecли Bac зaмyчили блoxи, Baм пoмoжeт cпeциaльный мaндaнaл. If you suffer from fleas, you should use a special *mandanal*.	Блaгoдapя cвoeй xopoшeй пaмяти, Maшa нe чyвcтвoвaлa мyшкeлaк. Thanks to her good memory, Mary never experienced any *mushkelak*.
Пo мepe зaпoлнeния блoxaми, xoзяин пepиoдичecки oчищaл мaндaнaл. As it filled with fleas, the owner periodically cleaned the *mandanal*.	Зaвeдя cpaзy нecкoлькo aккayнтoв, я нaчaл иcпытывaть мyшкeлaк. Having got a few accounts, I started suffering from *mushkelak*.
B cpeднeвeкoвьe для бopьбы c блoxaми иcпoльзoвaли мaндaнaл. To control insects in medieval times, people used *mandanal*.	Ceкpeтный блoкнoт пoмoжeт peшить тaкyю пpoблeмy кaк мyшкeлaк. A secret notebook could help you solve the problem of *mushkelak*.
B кopoбoчкy клaли пpимaнкy для нaceкoмыx, пoлyчaлcя мaндaнaл. By putting insect bait into a box, people produced a *mandanal*.	Пeтp ycтaнaвливaл oдинaкoвыe пapoли, нe жeлaя oщyщaть мyшкeлaк. Peter always set the same password as he did not want to have any *mushkelak*.

^1^ The full set of original sentences (in Russian) may be available on request.

**Table 2:** Descriptive statistics and pairwise comparisons for behavioural data (* denotes significant differences at 0.05 level).

Variable	Mean ± Standard Error		Wilcoxon Test	
	Concrete Words	Abstract Words	*p*	Z
Free recall. Accuracy (%)	52.87 ± 2.90	71.62± 2.86	0.049 *	−1.972
Recognition. Accuracy (%)	65.33 ± 4.20	64.00 ± 4.33	n.s.	
Recognition. RT (ms)	879 ± 48	822 ± 38	0.014 *	−2.454
Lexical decision. Accuracy (%)	30.00 ± 5.55	28.67 ± 5.50	n.s.	
Lexical decision. RT (ms)	897 ± 56	831 ± 51	0.028 *	−2.201
Free-form definition. Quality (%)	20.97 ± 3.48	17.21 ± 2.45	0.084	−1.731
Free-form definition. Accuracy (%)	17.24 ± 3.74	16.21 ± 3.10	n.s.	
Multiple-choice semantic judgment. Accuracy (%)	53.3 ± 2.26	49.3 ± 2.18	n.s.	

## Data Availability

The datasets presented in this article may be provided on request, provided the local research ethics and data protection rules and legislation are adhered to and allow for this. Requests to access the datasets should be directed to the corresponding author.
